# Traditional Chinese Medicine in the Treatment of Patients Infected with 2019-New Coronavirus (SARS-CoV-2): A Review and Perspective

**DOI:** 10.7150/ijbs.45538

**Published:** 2020-03-15

**Authors:** Yang Yang, Md Sahidul Islam, Jin Wang, Yuan Li, Xin Chen

**Affiliations:** State Key Laboratory of Quality Research in Chinese Medicine, Institute of Chinese Medical Sciences, University of Macau, Macau SAR 999078, China

**Keywords:** SARS-CoV-2, Traditional Chinese Medicine (TCM), coronavirus pneumonia

## Abstract

Currently, Severe Acute Respiratory Syndrome Coronavirus 2 (SARS-CoV-2, formerly known as 2019-nCoV, the causative pathogen of Coronavirus Disease 2019 (COVID-19)) has rapidly spread across China and around the world, causing an outbreak of acute infectious pneumonia. No specific anti-virus drugs or vaccines are available for the treatment of this sudden and lethal disease. The supportive care and non-specific treatment to ameliorate the symptoms of the patient are the only options currently. At the top of these conventional therapies, greater than 85% of SARS-CoV-2 infected patients in China are receiving Traditional Chinese Medicine (TCM) treatment. In this article, relevant published literatures are thoroughly reviewed and current applications of TCM in the treatment of COVID-19 patients are analyzed. Due to the homology in epidemiology, genomics, and pathogenesis of the SARS-CoV-2 and SARS-CoV, and the widely use of TCM in the treatment of SARS-CoV, the clinical evidence showing the beneficial effect of TCM in the treatment of patients with SARS coronaviral infections are discussed. Current experiment studies that provide an insight into the mechanism underlying the therapeutic effect of TCM, and those studies identified novel naturally occurring compounds with anti-coronaviral activity are also introduced.

## Introduction

In December 2019, there was an outbreak of unexplainable pneumonia in Wuhan city, Hubei province, China 1. By Jan 7, 2020, it was confirmed that a new type of coronavirus named SARS-CoV-2 (formerly named as 2019-nCoV) had emerged 2. The World Health Organization (WHO) named the Wuhan pneumonia as Coronavirus Disease-2019 (COVID-19) on Feb 11, 2020 3. The COVID-19 patients showed typical respiratory symptom (such as cough, fever, and lung damage) and some other symptoms such as fatigue, myalgia, and diarrhea 4, 5. As of February 17, 2020, a total of 73,332 cases of the SARS-CoV-2 infected pneumonia has been reported in China and 25 other countries, of which 72,528 cases was found in China 6. Due to the rapid spread of SARS-CoV-2 through human-to-human transmission, the cases currently continue to rise. SARS-CoV-2 extracted from patients with pneumonia in Wuhan is an enveloped single stranded RNA-type beta-coronavirus 7. The genome sequences of SARS-CoV-2 shared 79.5% sequence identity to severe acute respiratory syndrome-related coronaviruses (SARS-CoV) 8, 9. In addition, the spike (S) protein of SARS-CoV-2 and SARS-CoV enters human alveolar epithelial cells through binding angiotensin- converting enzyme 2 (ACE2) receptor 8.

COVID-19 can be diagnosed by either chest CT radiography or a laboratory testing. Unfortunately, specific antiviral drugs or vaccines currently have not been available for the treatment 10, 11. According to the current clinical guideline in China and the experiences in the treatment of SARS or Middle East Respiratory Syndrome (MERS) patients, both conventional medicine and traditional Chinese medicine (TCM) are used for the treatment of patients with infection of SARS-CoV-2 in China 12-14. This review mainly focuses on the discussion of TCM usage in the treatment of COVID-19 patients, in the context of current conventional management. Due to the homology in epidemiology, genomics, and pathogenesis of the SARS-CoV-2 and SARS-CoV 8, 9, and widely usage of TCM in the treatment of patients infected with SARS-CoV in 2002-2003 15, the clinical evidence showing the efficacy and safety of TCM in the treatment of patients with the emerging coronaviral will be summarized and analyzed, including the laboratory studies that provide an insight into molecular basis of therapeutic benefits.

## Conventional treatment of SARS-CoV-2: is there a room for Chinese medicine?

Due to the absence of a specific antiviral therapeutics and vaccine, main treatment strategy for COVID-19 is supportive care, which is supplemented by the combination of broad-spectrum antibiotics, antivirals, corticosteroids and convalescent plasma 16 (Table 1). HIV protease inhibitors ritonavir and lopinavir have been used, typically in combination with appropriate antibiotics or with IFNα-2b, in the treatment of SARS-CoV-2 infected patients 7, 17. Nucleoside analogs such as ribavirin 12 may be potentially beneficial for the treatment of COVID-19, since ribavirin was approved for treating respiratory syncytial virus (RSV) infection 18 and used extensively during the SARS and MERS outbreak 10. However, ribavirin had severe side effects such as anemia 18 and whether it had sufficient antiviral activity against SARS-CoV-2 is unclear. Nucleoside analogs favipiravir (T-705) can effectively inhibit the activity of RNA polymerase of RNA viruses such as influenza 19. A recent *in vitro* study found that it had the anti-SARS-CoV-2 activity 20, but the *in vivo* effect remains elusive. Remdesivir may be the most promising antiviral drug for treating COVID-19. It has *in vitro* and *in vivo* antiviral activity against a wide array of RNA viruses including SARS and MERS 21, and could decrease viral loads and pathology of lungs in animal models 22. A study showed remdesivir markedly inhibited the infection of SARS-CoV-2 in Vero E6 cells 20, and most symptoms of the first US patient infected with SARS-CoV-2 had resolved swiftly after intravenous administration with remdesivir 23. Currently, it is under clinical trial to evaluate the safety and efficacy of intravenous remdesivir for patients with SARS-CoV-2 infection 24. Oral oseltamivir has been used for the treatment of the cases with SARS-CoV-2 7, while its efficacy currently remains uncertain.

Host-targeted small molecules approved for other human diseases may modulate the virus-host interactions of SARS-CoV-2. Chloroquine, a potential broad-spectrum antiviral drug 25, 26, was shown by a recent study had anti-SARS-CoV-2 activity 20. Its clinical efficacy is under study in an open-label trial (ChiCTR2000029609) 12. IFNα (5 million U) atomization inhalation was recommended as antiviral therapy to treat SARS-CoV-2 16. A trial testing IFNα-2b combination of the approved anti-HCV inhibitors has been initiated 17, however, whether it could act synergistically against SARS-CoV-2 is unclear.

Corticosteroids were frequently used to suppress the elevated cytokine levels in patients with SARS-CoV 27, 28 and MERS-CoV 29, 30. However, there are no evidence showing that the mortality of SARS and MERS patients was reduced by the treatment with corticosteroids, while the clearance of viral was delayed by such treatment 31-33. Consequently, corticosteroids are not suggested to systemically use in SARS-CoV-2 infected patients 34, 35.

Previously, it was shown that, either in severe influenza or SARS-CoV infection, convalescent plasma treatment could significantly decrease viral load and reduce the mortality 31, 36. Convalescent plasma has been used for severe SARS-CoV-2 infection in China 22, although promising, the efficacy and safety need to be carefully further evaluated.

Consistent with previous analysis, WHO also concluded "to date, there is no specific medicine recommended to prevent or treat SARS-CoV-2" 37. TCM has been used in control of infectious diseases for thousands of years. There is a clear room for the intervention of TCM as a complementary therapy for COVID-19 patients. It is reported that the patients with SARS-CoV infection have benefited from TCM treatment 38, including amelioration of side effect of conventional therapeutics 39, 40. Based on these factors, there is a general expectation that TCM would be a valuable weapon in the armory against SARS-CoV-2.

## Traditional Chinese Medicine in the treatment of patients infected with SARS-CoV: clinical evidence

Application of TCM in the treatment of SARS-CoV-2 is largely inspired by the treatment of SARS caused by outbreak of SARS coronavirus (SARS-CoV) in the late of 2002 in the Guangdong Province of China which spread rapidly during the 2003, with the cumulative number worldwide of over 8,000 41-43. Ranging from case reports, case series, controlled observational studies and randomized clinical trials, clinical studies aiming to examine the effect of TCM on SARS have been carried out and reported. There are quite compelling evidences support the notion that TCM has beneficial effect in the treatment or prevention of SARS. For example, the rate of fatality in Hong Kong and Singapore was approximately 18%, while the rate for Beijing was initially more than 52% until the 5^th^ of May and decreased gradually to 4%-1% after the 20^th^ of May in 2003. The dramatic reduced fatality from late May in Beijing was believed to be associated with the use of TCM as a supplement to the conventional therapy 44. Lau and colleagues reported that, during SARS outbreak, 1063 volunteers including 926 hospital workers and 37 laboratory technicians working in high-risk virus laboratories used a TCM herbal extract, namely *Sang Ju Yin* plus *Yu Ping Feng San*. Compared with the 0.4% of infection in the control group, none of TCM users infected. Furthermore, there was some evidence that *Sang Ju Yin* plus *Yu Ping Feng San* could modulate T cells in a manner to enhance host defense capacity 45, 46. In a controlled clinical study, the supplementary treatment with TCM resulted in marked improvement of symptoms and shortened the disease course 47. The clinical beneficial effect of TCM appears to be supported by laboratory studies. For example, a high-profile research published in the *Lancet* reported that glycyrrhizin, a major active constituent *liquorice root* which is the most frequently used Chinese herb, potently inhibited the replication of clinical isolates of SARS virus 48. Another independent study confirmed the antivirus activity of glycyrrhizin by plaque reduction assays and this study found that another Chinese herbal compound baicalin also had the anti-SARS activity 49. Furthermore, Wang *et al*. found MOL376, a compound derived from TCM, may become a lead compound for SARS therapy by inhibition of cathepsin L, a target for the treatment of SARS 50.

There is a myriad of literature on TCM treatments for SARS published after the SARS epidemic in China. A critical analysis of these publications would be useful to confirm the beneficial effect of TCM. Liu *et al.* systematically reviewed eight randomized controlled trials, and concluded that, by combination with conventional medicine, TCM showed the beneficial effects such as decrease of mortality and relief of symptom, as well as control of fungal infections in patients with SARS. However, the evidence is not sufficient enough due to the poor quality of methodology used in the trials 13. Leung analyzed 90 peer-reviewed papers with reasonable quality from 130 publications and concluded that TCM used together with conventional treatment had some positive effects, including better control of fever, quicker clearance of chest infection and other symptoms. However, such beneficial effect of TCM is not conclusive and more high-quality clinical studies are required 15. In another thorough literature analysis, Liu and colleagues concluded that there was no benefit of adjuvant treatment with TCM in terms of mortality 39. Due to the lack of high quality TCM trials and biases that influenced the validity of results, Wu and colleagues suggested to re-run clinical trials of TCM for the treatment of acute respiratory tract infections (ARTIs) 51.

## Identification of anti-novel coronaviral compound from Traditional Chinese Medicine

Natural products used in TCM remains to be a wealthy source for the identification of novel therapeutic agents for the treatment of human diseases 52. In the past decade, scientists have made a considerable effort to identify multiple component herbal formulae in TCM with anti-SARS-CoV activity (Table 2). Further identification of chemical entities contained in TCM herbs responsible for the anti-SARS- CoV effect was also pursued (Table 3). Due to the homology of SARS-CoV and SARS-CoV-2, these previous studies may shed light on the naturally occurring compounds with the capacity to inhibit SARS-CoV-2.

3- chymotrypsin-like protease (3CLpro) is vital for replication of virus, and thus represents a promising drug target for the development of therapeutics agents for SARS-CoV as well as other human coronaviruses including SARS-CoV-2. It was reported that following TCM herbal extracts had the capacity to inhibit the enzymatic activity of SARS 3CLpro: Chinese *Rhubarb* extracts (IC50: 13.76 ± 0.03 μg/mL) 53, water extract of *Houttuynia cordata* 54, 55*,* flavonoid extracted from* litchi* seeds 56 and beta-sitosterol (IC50: 1210µM) extracted from the root extract of *Isatis indigotica* 57. Further, following herb-derived naturally occurring compounds including sinigrin (IC50: 217µM), indigo (IC50: 752µM), aloe-emodin (IC50: 366 µM), hesperetin (IC50:8.3 µM) 57, quercetin (IC50: 73µM), epigallocatechin gallate (IC50: 73µM), gallocatechin gallate (IC50: 47 µM) 58, herbacetin, rhoifolin and pectolinarin 59 were able to inhibit the SARS 3CLpro activity. Moreover, the flavonoids namely herbacetin, isobavaschalcone, quercetin 3‐β‐D‐glucoside, and helichrysetin had the potential to block the enzymatic activity of MERS‐CoV 3CL protease 60.

The helicase protein is also considered as a potential target for the development of anti-HCoV (human coronavirus) agents. Yu *et al.* reported scutellarein and myricetin potently inhibited the nsP13 (SARS-CoV helicase protein) *in vitro* by affecting the ATPase activity 61. The RNA- dependent RNA polymerase (RdRp), a key enzyme responsible for both positive and negative-strand RNA synthesis, also represents another potential druggable target. It was shown that the extracts of *Kang Du Bu Fei Tang* (IC50:471.3 µg/mL), *Sinomenium acutum* (IC50:198.6 µg/mL), *Coriolus versicolor* (IC50:108.4 µg/mL) and *Ganoderma lucidum* (IC50:41.9 µg/mL) inhibited SARS-CoV RdRp in a dose- dependent manner 54. Wu *et al.* performed large- scale screening of existing drugs, natural products, and synthetic compounds (>10000 compounds) to identify effective anti-SARS-CoV agents through a cell-based assay with SARS virus and Vero E6 cells 62. They found that ginsenoside-Rb1 isolated from *Panax ginseng*, aescin isolated from the horse chestnut tree, reserpine contained in the genus *Rauwolfia* and extracts of *eucalyptus* and *Lonicera japonica* inhibited SARS-CoV replication at non-toxic concentrations 62.

Same as SARS-CoV and HCoV-NL63, SARS-CoV-2 uses host receptor ACE2 for the cellular entrance 63-66. Therefore, TCM with the capacity to target ACE2 holds the promise to prevent the infection of SARS-CoV-2. Emodin from genus *Rheum* and *Polygonum* 67, baicalin from in *Scutellaria baicalensis* 44, 68, nicotianamine from foodstuff (especially “soybean ACE2 inhibitor (ACE2iSB)”) 69, scutellarin 70, tetra-*O*-galloyl-β-D-glucose (TGG) from *Galla chinensis* and luteolin from *Veronicalina riifolia* 71 markedly inhibited the interaction of SARS-CoV S-protein and ACE2. However, the anti-SARS-CoV activity of these compounds remain to be evaluated. In addition, inhibition of the 3a ion channel by emodin 72 or kaempferol derivatives- juglanin 73 could potentially prevent the viral release from the infected cells. Saikosaponins 74, glycyrrhizin 48, 75, quercetin and TSL-1 extracted from *Toona sinensis* Roem 76 purportedly had potent anti-SARS-CoV effects by inhibition of viral cellular entry, adsorption, and penetration.

Overwhelming inflammatory responses are attributable to the deaths of patients with infection of SARS-CoV, or MERS-CoV, or COVID-19. Thus, anti-inflammatory agents presumably could reduce the severity and mortality rate 77. *Shuang Huang Lian,* a TCM herbal product prepared from *Lonicerae japonicae* Flos, *Scutellariae radix* and *Fructus Forsythiae,* purportedly had the activity to inhibit SARS-CoV-2 78. Interestingly, We have shown that this herbal preparation potently inhibited staphylococcal toxic shock syndrome toxin 1 (TSST-1)-induced production of cytokines (IL-1β, IL-6, TNF-α, IFN-γ) and chemokines (MIP-1α, MIP-1β and MCP-1) by peripheral blood mononuclear cell (PBMC) 79. In line with our results, this herbal product was shown to markedly reduced the transcriptional and translational levels of inflammatory cytokines TNF-α, IL-1β, and IL-6 in lipopolysaccharide-stimulated murine alveolar macrophages 80. Indirubin is an active ingredient of a TCM preparation *Dang Gui Long Hui Pill,* had strong antiviral and immunomodulatory effects, as shown by a study based on the observation of influenza H5N1 virus-infected human macrophages and type-I alveolar epithelial cells 81. *Lian Hua Qing Wen Capsule* was reported to have *in vitro* activity in inhibition of propagation of various influenza viruses. This TCM herbal product not only blocked the early stages of influenza virus infection but also inhibited virus-induced gene expression of IL-6, IL-8, TNF-a, IP-10, and MCP-1 82. Additionally, a study by Dong *et al*. reported that the levels of IL-8, TNF-α, IL-17, and IL-23 in the sputum and of IL-8 and IL-17 in the blood were markedly decreased after *Lian Hua Qing Wen Capsule* treatment in patients with acute exacerbation of chronic obstructive pulmonary disease 83. A self-control study by Poon *et al.* showed that the administration of the TCM herbal formulas (*Sang Ju Yin* and *Yu Ping Feng San*) may have beneficial immunomodulatory effects for the prevention of viral infections including SARS-CoV 46.

Moreover, a number of anti-coronaviral agents have been identified from TCM herbs, although the mechanisms of action have not yet been elucidated. For example, extracts from *Lycoris radiata*, *Artemisia annua*, *Pyrrosia lingua*, and* Lindera aggregate* possessed the anti-SARS‑CoV activity 84, 3β-Friedelanol isolated from *Euphorbia neriifolia* 85, Blancoxanthone isolated from the roots of *Calophyllum blancoi* 86 exhibited anti-HCoV-229E activity.

## Traditional Chinese Medicine used in the treatment of SARS-CoV-2-infected patients: the current situations

TCM is highly valued by the government of China in their campaign to contain and eradiate SARS-CoV-2. For example, Health Commission in 26 provinces have officially declared that TCM should be used in combination with conventional medicine in the treatment of COVID-19 patients. On 17, February, National Health Commission (NHC) of the People's Republic of China reported that 60,107 confirmed COVID-19 patients (85.20% of total confirmed cases) had been treated with TCM 87. As for March 1, 2020, a total of 303 ongoing clinical trials aiming to evaluate the efficacy and safety of treatments for CoV-19 patients have been launched in China. Among them, 50 trials (16.5%) are about the use of TCM, including 14 cases (4.6%) to examine the effect of combined treatment with TCM and Western medicine. In 22 TCM trials (7.3%), the effect of self-made herbal preparations such as *Xin Guan-1 Formula*, *Xin Guan-2 Formula* and *Qing Yi-4* are examined. In another 14 TCM trials (4.6%), commercially available TCM products such as *Tan Re Qing Injection* and* Lian Hua Qing Wen Capsule* are studied (Table 4).

To date, NHC has published 6 editions Guidelines of Diagnosis and Treatment for COVID-19 88. Since the fourth versions, different herbal medicines used in TCM system has been recommended for the treatment of COVID-19, based on the stage of disease and symptom differentiation 89. According to the latest edition of Guideline 88, following multiple component Chinese herbal products are recommended for the patients in the medical observation period, presumably as a preventive measure: *Huo Xiang Zheng Qi Shui*, *Lian Hua Qing Wen Capsule*, S*hu Feng Jie Du Capsule* and *Jin Hua Qing Gan Granule*. In the clinical treatment period, *Qing Fei Pai Du Tang*, *Xi Yan Ping Injection*, *Xue Bi Jing injection*, *Re Du Ning Injection, Tan Re Qing Injection*, *Xing Nao Jing Injection* and some other Chinese medicine formulae should be selected 90. In addition, for the patients in critical condition, *Shen Fu Injection*, *Sheng Mai Injection*, *Shen Mai Injection*, *Su He Xiang Pill* and *An Gong Niu Huang Pill* should be administered (Table 5).

Through analysis of the frequency of TCM used in 23 provinces, Luo, et al. 37 concluded that *Astragalus membranaceus, Glycyrrhizae uralensis, Saposhnikoviae divaricata, Rhizoma Atractylodis Macrocephalae, Lonicerae Japonicae Flos, Fructus forsythia, Atractylodis Rhizoma, Radix platycodonis, Agastache rugosa*, and *Cyrtomium fortune J. Sm* were 10 most commonly used Chinese herbs in the treatment of COVID-19. Xu, *et al.* 91 reported that *Astragalus membranaceus* and *Yu Ping Feng* were used in the 13 prevention programs (in Beijing, Tianjin, et al.) for “reinforcing vital *qi*”, a terminology used in TCM that is similar to boosting host defense capacity. *Ophiopogon japonicas* and *Scrophularia ningpoensisand* are TCM herbs which were most frequently used for “nourishing *yin*” in northern China, while *Atractylodis Rhizoma, Agastache rugosa* and other Chinese medicinal herbs with the property of “aromatic dehumidification” were commonly used in southern China (Table 6).

According to the report of National Administration of Traditional Chinese Medicine, up to February 5th, 2020, 214 COVID-19 patients were treated with *Qing Fei Pai Du Tang* in Shanxi, Hebei, Heilongjiang and Shaanxi Provinces with overall effective rate ≥ 90%. Among them, the symptoms of majority of patients (≥60%) were markedly improved, while illness of others (30%) was stabilized 92. After that, 701 COVID-19 patients were treated with *Qing Fei Pai Du Tang* in 10 provinces in China. The result showed that 130 patients (18.5%) were completely cured after treatment. The treatment also resulted in the disappearance of characteristic symptoms of COVID-19 such as fever and cough in 51 patients (7.27%). In addition, symptom improvement or stabilization were observed in 268 patients (38.2%), and in 212 patients (30.2%), respectively 87. Yao, *et al*. and Lu, *et al.* 93, 94 retrospectively analyzed the clinical efficacy of *Lian Hua Qing Wen Capsule* in treatment of confirmed and suspected COVID-19 patients. The results indicated that this herbal product could markedly relieve major symptoms such as fever and cough and had the capacity to promote the recovery.

Some patients with mild illness in the early stage could suddenly progress to severe disease, and eventually died due to septic shock with multiple organ dysfunction syndrome (MODS), which was associated with cytokine storm 95. There is compelling evidence that some TCM herbal products or its components have potent immunosuppressive effects, as shown by our own and other's studies 79, 96-103. For example, Wang, *et al*. 104 reported that *Shen Fu Injection* could inhibit the lung inflammation and decrease the levels of IL-1β, IL-6 and other cytokines. Chang, *et al*. 105 reported that *Re Du Ning Injection* could markedly reduce the levels of IL-1β, TNF-α, IL-8, IL-10, and some other cytokines of LPS-induced model of acute lung injury in rats. We recently reported that tetrandrine, a compound isolated from an anti-rheumatic Chinese herb, could potently inhibit proinflammatory Th1, Th2 and Th17 responses in LPS-challenged mice 106. Therefore, TCM with the capacity to inhibit cytokine storm and its devastating consequences may be harnessed in the treatment of severe COVID-19 patients.

Currently, the laboratory study on the effect of TCM is apparently lagging behind the clinical application of TCM in the treatment of COVID-19 patients. Nevertheless, some scientists have started to examine the effect of TCM products or its components on SARS-CoV-2 in their laboratories. For example, an *in vitro* study showed that S*huang Huang Lian Oral Liquid* had the inhibitory effect on SARS-CoV-2 78. However, its clinical efficacy and safety for the treatment of COVID-19 patients has not been evaluated. We noticed that this TCM product was not recommended by HNC's Guideline 89. Same as SARS-CoV, SARS-CoV-2 uses receptor ACE2 for the cellular entrance 8. Theoretically, blockade of ACE2 can prevent the infection of SARS-CoV-2. Chen and Du thus performed the molecular docking study and they found that TCM-derived compounds, including as baicalin, scutellarin, hesperetin, glycyrrhizin and nicotianamine could interact with ACE2 107. Therefore, these compounds as well as herbs containing these ingredients may have the capacity to inhibit the infection of SARS-CoV-2. We anticipate more experiment studies showing anti-SARS-CoV-2 activity of TCM or its components will be published in the near future.

## Closing remarks

TCM has accumulated thousand-of-year's experiences in the treatment of pandemic and endemic diseases. Providing complementary and alternative treatments are still urgently needed for the management of patients with SARS-CoV-2 infection, experiences in TCM is certainly worth learning. Fighting against current epidemics also provide an opportunity to test the true value of TCM in treating emerging contagious diseases. Randomized, double-blind and placebo-controlled studies is the best way to provide the most reliable evidence for a therapy, including TCM. It is encouraging that the controlled clinical studies to evaluate the efficacy of TCM in the treatment of SARS-CoV were conducted and reported. However, the most of these studies were found to be poorly designed and the results could lead to potential biases in evaluating the effectiveness of TCM treatment 13. Hopefully, current clinical study to evaluate the effect of TCM on COVID-19 will use more strict protocols, concealment of allocation, and double-blinding, in order to ensure the compliance of international acceptable standards. Furthermore, standardized products of TCM, rather than self-prepared formulations, should be used in clinical study. Experiment study may be able to elucidate the mechanism underlying the therapeutic effect of TCM in the treatment of COVID-19. The further study of TCM may lead to the identification of novel anti human coronavirus compounds that may eventually prove to be useful in the treatment of SARS-CoV-2 or other emerging fatal viral diseases as conventional therapeutic agents.

The safety of TCM in the treatment of emerging coronavirus diseases was not included in the observation on SARS patients 13. It was reported that some herbs used in TCM contain nephrotoxins and mutagens 108, while the toxicological features of the most of Chinese herbal medicines remain to be fully understood 109. Furthermore, herbs used in TCM can mimic, or magnify, or oppose the effect of conventional medicines 110. Thus, the safety of TCM used in treatment of emerging coronavirus infections should be carefully evaluated. It is particularly important to avoid toxicity or interfere with the efficacy of conventional treatment caused by herb-drug interaction.

## Supplementary Material

Supplementary figures and tables.

## Figures and Tables

**Table 1:** Conventional treatment of patients with SARS-CoV-2 infection

Type of treatment	Therapeutic agent or device	Reference
**Oxygen therapy**	Nasal cannula Non-invasive mechanical ventilation Invasive mechanical ventilation ECMO*	16
**Antibiotics combination**	Amoxicillin Azithromycin Fluoroquinolones	16
**Antivirals**	Lopinavir/ ritonavir	16, 17
	Ribavirin	16, 18
	Favipiravir (T-705)	19, 20
	Remdesivir	20-23
	Oseltamivir	7
	Chloroquine	20, 36
	Interferon	7, 17
**Corticosteroids**	Methylprednisolone	7
**Convalescent plasma**	Convalescent plasma	22

*ECMO, extracorporeal membrane oxygenation.

**Table 2:** TCM herb formulae used for the Treatment of SARS-CoV infection

TCM Formula	Composition	Therapeutics effect	Reference
*Yin Qiao San*	*Fructus Forsythiae, Flos Lonicerae, Radix Platycodonis, Herba Menthae, Herba Lophatheri, Radix Glycyrrhizae, Herba Schizonepetae,* Fermented soybean*, Fructus arctii,* and* Rhizoma Phragmitis*	“Disperses wind-heat, clears heat, and relieves toxicity”, according to TCM theory Treatment of upper respiratory tract infection. Improvement of the function of upper respiratory mucosal immune system	111, 112
*Yu Ping Feng San*	*Astragali radix, Astragalus membranaceus, Atractylodes macrocephala, and Saposhnikoviae Radix*	“Tonifying *qi*” to protect from external pathogens”, according to TCM theory Reportedly have antiviral, anti-inflammatory and immunoregulatory effects	113-115
*Sang Ju Yin* and *Yu Ping Feng San*	*Sang Ju Yin* [made with *chrysanthemum, mulberry leaf*, and 6 other herbs] and *Yu Ping Feng San*	Reportedly have anti-viral and immunoregulatory effects	46
*Lian Hua Qing Wen Capsule*	*Forsythia suspensa*, *Ephedra sinica, Lonicera japonica, Isatis indigotica, Mentha haplocalyx, Dryopteris crassirhizoma, Rhodiola rosea, Gypsum Fibrosum*, *Pogostemon cablin, Rheum palmatum* , *Houttuynia cordata, Glycyrrhizae, uralensis,* and* Armeniaca sibirica*	“Clear heat and detoxify, removes lung hotness”, according to TCM theory Reportedly have antiviral, anti-inflammatory and immunoregulatory effects.	82, 83
S*huang Huang Lian*	*Lonicera japonica, Scutellaria baicalensis,* and *Forsythia suspensa*	“Clear heat and detoxify, remove lung hotness”, according to TCM theory Reportedly has anti-SARS-CoV-2 activity Reportedly has immunosuppressive effects	78, 80, 116
*Ma Xin Gan Shi Tang*	*Ephedrae herba, Armeniacae semenamarum*), *Glycyrrhizae radix et rhizome, Gypsum fibrosum,* and *Da Yuan Yin [Arecae semen, Magnoliae officinalis cortex, Tsaoko fructus,Anemarrhenae rhizoma, Dioscoreae rhizoma, Scutellariae radix,* and* Glycyrrhizae raadix et rhizome]*	“Facilitate the flow of the lung “*qi”* and clear away heat”, according to TCM theory Reportedly have anti-SARS-CoV activity	117, 118

**Table 3:** TCM herbal extracts or TCM-derived Compounds with anti-HCoV Activity

TCM Compound (s)	Mode of action	Reference
Plant-derived phenolic compounds and Root extract of *Isatis indigotica*	Inhibit the cleavage activity of SARS-3CLpro enzyme	57
Water extract of *Houttuynia cordata*	Inhibit the viral SARS-3CLpro activity Block viral RNA‑dependent RNA polymerase activity (RdRp) Immunomodulation	54, 55
Scutellarein and myricetin	Inhibit nsP13 by affecting the ATPase activity	61
Glycyrrhizin from *Glycyrrhizae radix*	Inhibit viral adsorption and penetration	48, 75
Herbacetin, quercetin, isobavaschalcone, 3‐β‐D‐glucoside and helichrysetin	Inhibit cleavage activity of MERS-3CLpro enzyme	60
Tetrandrine, fangchinoline, and cepharanthine	Inhibit the expression of HCoV- OC43 spike and nucleocapsid protein. Immunomodulation	106, 119
Chinese Rhubarb extracts	Inhibit SARS-3CLpro activity	53
Flavonoids (For example: extracted from litchi seeds, herbacetin, rhoifolin, pectolinarin, quercetin, epigallocatechin gallate, and gallocatechin gallate)	Inhibit SARS-3CLpro activity	56, 58, 59
Quercetin and TSL-1 from *Toona sinensis* Roem	Inhibit the cellular entry of SARS-CoV	76
Emodin derived from genus *Rheum* and *Polygonum*	Inhibit interaction of SARS-CoV Spike protein and ACE2 Inhibit the 3a ion channel of coronavirus SARS‐CoV and HCoV‐OC43	67, 72
Kaempferol derivatives	Inhibit 3a ion channel of coronavirus	73
Baicalin from *Scutellaria baicalensis*	Inhibit Angiotensin-converting enzyme (ACE)	44, 68
Saikosaponins	Prevent the early stage of HCoV‑22E9 infection, including viral attachment and penetration	74
Tetra-*O*-galloyl-β-D-glucose and luteolin, from *Galla chinensis* and* Veronicalina riifolia* respectively	Avidly binds with surface spike protein of SARS-CoV	71

**Table 4:** Ongoing TCM Clinical Trials for the treatment of SARS-CoV-2 infection

Registration number	Design type	Title	TCM herbal medicine	Sample size	Phase
ChiCTR2000029432	CCT	A real world study for the efficacy and safety of large dose Tanreqing Injection in the treatment of patients with novel coronavirus pneumonia (COVID-19)	*Tan Re Qing Injection*	72	4
ChiCTR2000029434	RCT	A randomized, open-label, blank-controlled trial for Lian-Hua Qing-Wen Capsule/Granule in the treatment of novel coronavirus pneumonia (COVID-19)	*Lian Hua Qing Wen Capsule/Granule*	400	4
ChiCTR2000029487	CCT	Clinical study for Gu-Biao Jie-Du-Ling in preventing of novel coronavirus pneumonia (COVID-19) in children	*Gu Biao Jie Du Ling*	200	0
ChiCTR2000029589	CCT	An open, prospective, multicenter clinical study for the efficacy and safety of Reduning injection in the treatment of ovel coronavirus pneumonia (COVID-19)	*Re Du Ning* *Injection*	60	0
ChiCTR2000029605	RCT	A randomized, open-label, blank-controlled, multicenter trial for Shuang-Huang-Lian oral solution in the treatment of novel coronavirus pneumonia (COVID-19)	*Shuang Huang Lian Oral Liquid*	400	4
ChiCTR2000029780	RCT	A multicenter, randomized, open, controlled trial for the efficacy and safety of Shen-Qi Fu-Zheng injection in the treatment of novel coronavirus pneumonia (COVID-19)	*Shen Qi Fu Zheng Injection*	160	4
ChiCTR2000029781	RCT	A multicenter, randomized, open and controlled trial for the efficacy and safety of Kang-Bing-Du granules in the treatment of novel coronavirus pneumonia (COVID-19)	*Kang Bing Du Granules*	160	4
ChiCTR2000029822	RCT	A randomized controlled trial for honeysuckle decoction in the treatment of patients with novel coronavirus (COVID-19) infection	*Jin Yin Hua Tang*	110	0
ChiCTR2000029991	RCT	A randomized, open-label, controlled trial for the safety and efficiency of Kesuting syrup and Keqing capsule in the treatment of mild and moderate novel coronavirus pneumonia (COVID-19)	*Ke Su Ting Syrup* */Ke Qing Capsule*	72	4
ChiCTR2000030043	RCT	Shen-Fu injection in the treatment of severe novel coronavirus pneumonia (COVID-19): a multicenter, randomized, open-label, controlled trial	*Shen Fu Injection*	300	4
ChiCTR2000030117	RCT	A multicenter, randomized, open, parallel controlled trial for the evaluation of the effectiveness and safety of Xiyanping injection in the treatment of common type novel coronavirus pneumonia (COVID-19)	*Xi Yan Ping Injection*	348	4
ChiCTR2000030255	RCT	Efficacy and safety of Jing-Yin Granule in the treatment of novel coronavirus pneumonia (COVID-19) wind-heat syndrome	*Jing Yin Granule*	300	4
ChiCTR2000030388	RCT	Efficacy and safety of Xue-Bi-Jing injection in the treatment of severe cases of novel coronavirus pneumonia (COVID-19)	*Xue Bi Jing Injection*	60	0
ChiCTR2000029813	RCT	Clinical Trial for Tanreqing Capsules in the Treatment of Novel Coronavirus Pneumonia (COVID-19)	*Tan Re Qing Capsules*	72	0

Notes: RCT: randomized controlled trial; CCT: controlled clinical trial.

**Table 5:** TCM recommended by 6th editions Guidelines of Diagnosis and Treatment for COVID-19 88.

Stage of disease	Symptom	Recommended Chinese patent medicine
**Medical observation period**	Fatigue with gastrointestinal discomfort	*Huo Xiang Zheng Qi Shui*
	Fatigue with fever	*Lian Hua Qing Wen Capsule, Shu Feng Jie Du Capsule, Jin Hua Qing Gan Capsule*
**Clinical treatment period** **(Confirmed patients)**	Mild cases	*Qing Fei Pai Du Tang*
	General cases	*Qing Fei Pai Du Tang*
	Several cases	*Xi Yan Ping Injection, Xue Bi Jing Injection, Re Du Ning Injection, Tan Re Qing Injection, Xing Nao Jing Injection, Qing Fei Pai Du Tang*
	Critical cases	*Xue Bi Jing Injection, Re Du Ning Injection, Tan Re Qing Injection, Shen Fu Injection, Sheng Mai Injection, Shen Mai Injection, Su He Xiang Pill, An Gong Niu Huang Pill*

**Table 6:** Frequently used TCM herbs for the Prevention of COVID-19 infection

Reported by	Herbs (Latin name)	Herbs (Chinese Pin Yin)	Applicable regions
**Luo, *et al*. 37**	*Astragalus membranaceus*	Huangqi	23 provinces covered Northeast, North, Central (including Wuhan), South, East, Northwest, and Southwest China.
	*Glycyrrhizae uralensis*	Gancao	
	*Saposhnikoviae divaricata*	Fangfeng	
	*Rhizoma Atractylodis Macrocephalae*	Baizhu	
	*Lonicerae Japonicae Flos*	Jinyinhua	
	*Fructus Forsythiae*	Lianqiao	
	*Atractylodis Rhizoma*	Cangzhu	
	*Radix platycodonis*	Jiegeng	
	*Agastache rugosa*	Huoxiang	
	*Cyrtomium fortune J. Sm*	Guanzhong	
**Xu, *et al.*^91^**	*Astragalus membranaceus*	Huangqi	Beijing, Tianjin, Shandong, Shaanxi, Gansu, Hebei, Shanxi, Henan, Hubei, Jiangxi, Hunan, and Yunnan
	*Atractylodis Rhizoma*	Cangzhu	Five regions in southern China (Hubei, Jiangxi, Hunan, Yunnan, and Wuhan)
	*Eupatorii Herba*	Peilan	
	*Agastache rugosa*	Huoxiang	
	*Ophiopogon japonicas*	Maidong	Eight regions in northern China (Beijing, Tianjin, Hebei, Henan, Shaanxi, Shanxi, Gansu, and Shandong)
	*Scrophularia ningpoensis*	Xuanshen	
	*Rhizoma phragmitis*	Lugen	
	*Adeinophora stricta Miq*	Shashen	
	*Dendrobium nobile Lindl.*	Shihu	
