# Supplementary material for: Traditional Chinese Medicine in the Treatment of Patients Infected with 2019-New Coronavirus (SARS-CoV-2): A Review and Perspective
Source: Int J Biol Sci. 2020 Mar 15;16(10):1708–17. doi: 10.7150/ijbs.45538 (PMC7098036; doi:10.7150/ijbs.45538)
Supplement: Supplementary file 1 — Supplementary figures and tables. [file ijbsv16p1708s1.pdf]

Supplementary Table 1. List of TCM patent medicines used in the treatment of COVID-19

Pinyin	Simplified Chinese	Classified Chinese
An Gong Niu Huang Pill	安宫牛黄丸	安宮牛黃丸
Dang Gui Long Hui Pill	当归龙荟丸	當歸龍薈丸
Gu Biao Jie Du Ling	固表解毒灵	固表解毒靈
Huo Xiang Zheng Qi Shui	藿香正气水	藿香正氣水
Jin Hua Qing Gan Granule	金花清感颗粒	金花清感顆粒
Jin Yin Hua Tang	金银花汤剂	金銀花湯劑
Jing Yin Granule	荆银颗粒	荊銀顆粒
Kang Bing Du Granules	抗病毒颗粒	抗病毒顆粒
Kang Du Bu Fei Tang	抗毒补肺汤	抗毒補肺湯
Ke Qing Capsule	咳清胶囊	咳清膠囊
Ke Su Ting Syrup	咳速停糖浆	咳速停糖漿
Lian Hua Qing Wen Capsule	连花清瘟胶囊	連花清瘟膠囊
Ma Xin Gan Shi Tang	麻杏甘石汤	麻杏甘石湯
Qing Fei Pai Du Tang	清肺排毒汤	清肺排毒湯
Qing Yi-4	清疫 4 号	清疫 4 號
Re Du Ning Injection	热毒宁注射液	熱毒寧注射液
Sang Ju Yin	桑菊饮	桑菊飲
Shen Fu Injection	参附注射液	參附注射液
Shen Mai Injection	参麦注射液	參麥注射液
Shen Qi Fu Zheng Injection	参芪扶正注射液	參芪扶正注射液
Sheng Mai Injection	生脉注射液	生脈注射液
Shu Feng Jie Du Capsule	疏风解毒胶囊	疏風解毒膠囊
Shuang Huang Lian Oral Liquid	双黄连口服液	雙黃連口服液
Su He Xiang Pill	苏合香丸	蘇合香丸

Tan Re Qing Injection	痰热清注射液	痰熱清注射液
Xi Yan Ping Injection	喜炎平注射液	喜炎平注射液
Xin Guan-1 Formula	新冠一号方	新冠一號方
Xin Guan-2 Formula	新冠二号方	新冠二號方
Xing Nao Jing Injection	醒脑静注射液	醒腦靜注射液
Xue Bi Jing Injection	血必净注射液	血必淨注射液
Yu Ping Feng San	玉屏风散	玉屏風散
